# Psychosocial predictors of attitude toward premarital sexual practice among university students, Ethiopia

**DOI:** 10.3389/fpsyg.2024.1369964

**Published:** 2024-11-13

**Authors:** Yordanos Yibeltal Yedemie

**Affiliations:** Department of Psychology, College of Education and Behavioral Sciences, Bahir Dar University, Bahir Dar, Ethiopia

**Keywords:** self-esteem, peer pressure, HIV/AIDS, psychosocial, attitude, premarital sex

## Abstract

**Objectives:**

Premarital sexual practice during adolescence may lead to different sexual and reproductive health problems, including HIV/AIDS. The objective of this study was to assess psychosocial predictors of attitudes toward premarital sex among Bahir Dar University students.

**Methods:**

A correlational design was used. A multi-stage sampling technique was used to select 375 respondents for the study, although the analysis was based on the 361 questionnaires that were returned from the field and found useful for analysis. Data was collected using a self-developed and modified instrument on standardized scales. The internal consistency of the Perception of Peer Pressure Scale was 0.83, while the self-esteem measuring scale had a reliability of 0.77. The internal consistency of the sexual attitude inventory scale was 0.97. The data were quantitatively analyzed using (inferential statistics) logistic regression, one sample t test, and an independent sample *t* test.

**Results:**

The findings of the study showed that all the psychological variables investigated were found to significantly predict premarital sexual relationships among the respondents. Male students with the experience of premarital sex have a higher level of self-esteem and exposure to peer pressure than their female counterparts. A study suggests that male students with the experience of premarital sex have a higher level of self-esteem and exposure to peer pressure than their female counterparts. Self-esteem negatively predicted attitudes toward premarital sex.

**Conclusion:**

Scaling up the level of school counseling, especially in the area of sexual adjustment and modification of the psychological variables investigated, was recommended.

## 1 Introduction

Adolescence is a vital and transitional stage of life, from childhood to adulthood that is marked by curiosity and adventures (Aleke et al., 2021) and often linked with increased risk-taking, including premarital sex (Liang et al., 2019). Premarital sexual activity is a common sexual exploration among adolescents (Yip et al., 2013), which usually involves behaviors such as multiple sexual partners (Teferra et al., 2015), inconsistent or non-condom use (UNAIDS, 2015), and consumption of pornography (Akililu et al., 2015). The rate of teenage pregnancy and the high level of indulgence in premarital sex among school children in this 21st century have become subjects of discourse (Ogbueghu, 2017). These children have been observed engaging in sexual activities without proper education, and as such, they are exposed and induced to unfamiliar risks and Sexually Transmitted Diseases (STDs) such as gonorrhea, syphilis, candidiasis, chlamydia, and Acquired Immune Deficiency Syndrome (AIDS) and so on (Ezeugwu and Ede, 2016).

**Figure 1:**
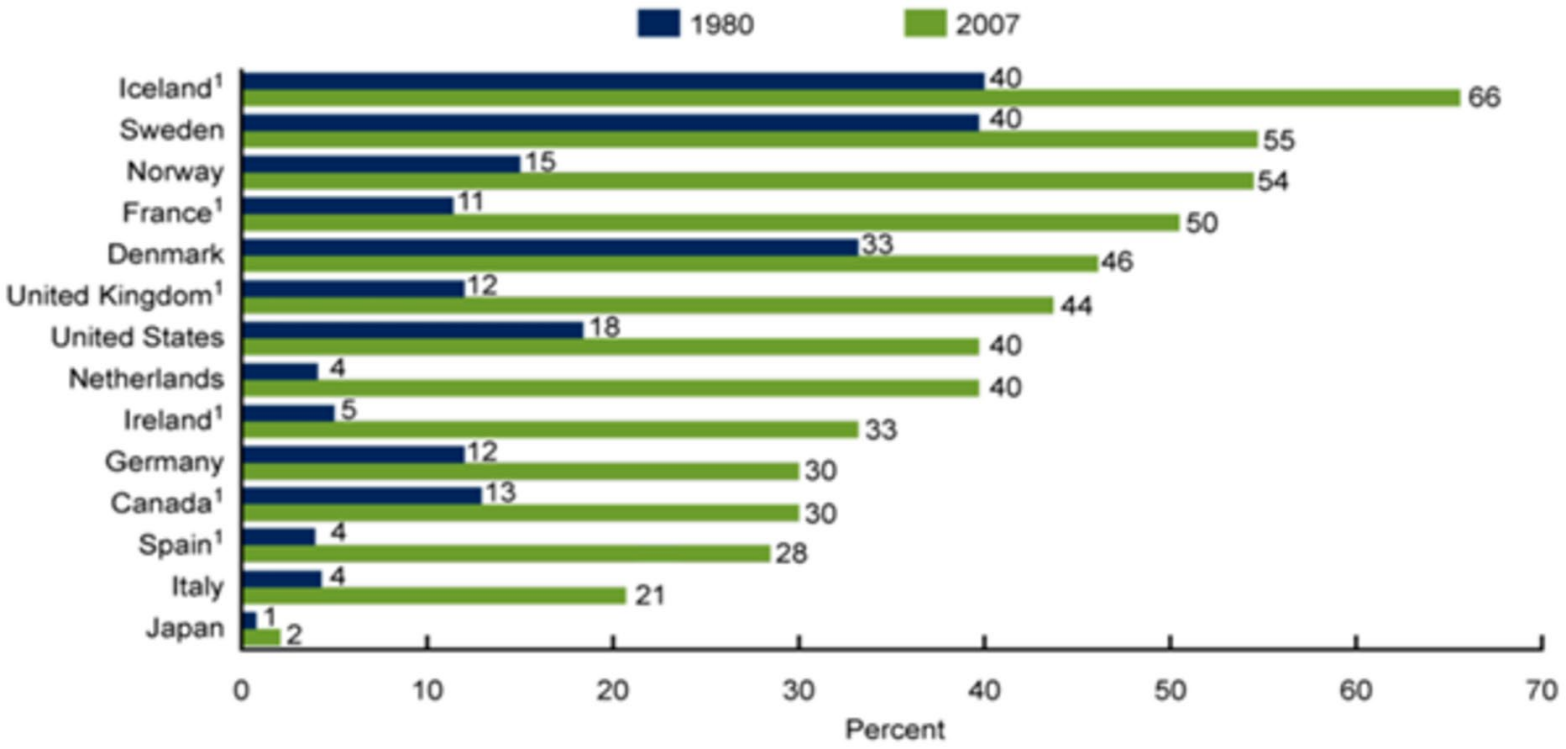
The practice of premarital sex in different countries.

Premarital sex, which is sexual intercourse between people who are not married, is becoming more common around the world (Akililu et al., 2015). It is risky because many young people lack the knowledge and guidance on how to prevent pregnancy and sexually transmitted infections (STIs) and human immunodeficiency virus (HIV) (Ezeugwu and Ede, 2016) (Figure 1).

Bhatta et al. (2016) suggest that sexual desire and arousal, sexual exploration, and the formation of a sexual identity are more evident in adolescence. These factors may be influenced by puberty, how one’s friends and family react to a more mature appearance, social norms about when and where to spend time with romantic partners, and cultural messages that shape one’s view of oneself as asexual being. Habtamu et al. (2015) define asexual fantasy as a mental image or thought that stimulates one’s sexuality and can enhance or create sexual arousal. A sexual fantasy exists only in one’s mind and can be generated by one’s imagination or memory.

According to Anene et al. (2017), premarital sex exposes university students to serious problems such as unsafe sex and emotional scars. However, universities are supposed to provide adequate information and formal education to adolescents, but premarital sexual practice among college students has been raising worldwide (Liang et al., 2019). Globally, 35.3 million people have HIV/AIDS, of which youths make up 2.1 million. Out of 2.3 million new HIV infections, more than half (15–24 years) are youths (Habtamu et al., 2015).

Premarital sex has negative consequences such as illegal abortions, HIV infections and school dropout in sub-Saharan Africa (Ogbueghu, 2017). One-fourth of the youths aged 15–19 had sex before 15 years old. Premarital sex is becoming more common in Ethiopia (Oljira et al., 2012). More than 25% of the school youths in Eastern Ethiopia and Lalibella Town had premarital sex (Berihun, 2014). In west Showa Zone, about 60% of the university youths had premarital sex (Endazenaw and Abebe, 2015).

Various scholars found different factors that influenced premarital sexual practice positively or negatively. Some of these factors are student’s age, sex, residence, education level, peer pressure, pocket money, substance use, alcohol drink, pornography movie, living arrangement, parent–child communication on sexual issues, peer influence, self-image, love and religious and life skill education (Ogbueghu, 2017).

Anene et al. (2017) stated that premarital sexual behaviors are more accepted in today’s society (Ogbueghu, 2017) with 69% of female adolescents and 64% of male adolescents aged 18–19 having had sexual intercourse even if they were not married (Ogbueghu, 2017), which shows some specific issues for unmarried sexually active people.

The world has seen an increase in the sexual activity of adolescence at younger age. Sub-Saharan Africa is the most affected region in the global AIDS epidemic. Sexual debut was reported as early as 12–13 years (Akililu et al., 2015). Therefore this study will examine the psychosocial factors that influence premarital sexual practice among first year university students by using peer pressure, self-esteem and attitude toward premarital sex as predictors.

The effects or consequences of premarital sex on adolescent students have been studied by many researchers, for example Akililu et al. (2015). This study explored the psychosocial factors that influence the attitude toward premarital sexual practice among first year students in Bahir Dar University. The research also aimed to find out the differences between male and female university students in their premarital sexual behavior.

The main purpose of this study was to focus on the factors that predict the attitude toward engaging in pre-marital sex, rather than its consequences that many studies have focused on Akililu et al. (2015). Female students are more at risk than male students because female students sometimes see sex as a way to express care, love and affection, with the hope of marriage in the future. They view sex as a sign of loyalty in a relationship. The consequences as shown by the study include unwanted pregnancy, STIs/HIV/AIDs, drug and alcohol use and abortion, all of which have biological effects. The psychological consequences include depression, phobia, guilt, regrets, bondage and stress. The social consequences are loss of family support, loss of self-esteem, damage of reputation, poor academic performance and isolation from peers, especially roommates (Akililu et al., 2015).

The report of Bahir Dar University Gender Office and Counseling Service Office stated that student school dropout was caused by premarital sex, relationship problems and unwanted pregnancy among youths in the study area. However, the prevalence of premarital sexual practice and its associated factors among university students in the study area had not been addressed yet. Therefore, this study aimed to identify the psychosocial factors that influence the attitude toward premarital sex among university youths in Bahir Dar University, Ethiopia.

**Figure 2:**
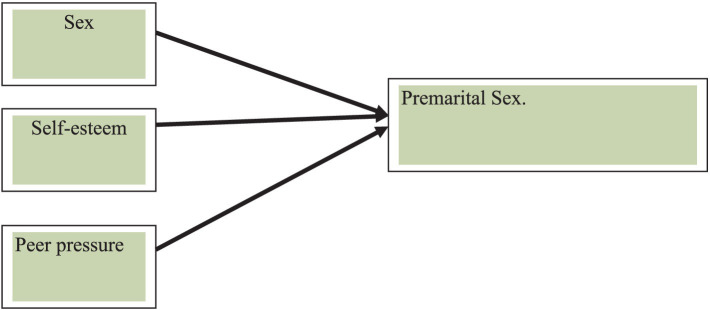
Psychosocial predictors of attitude toward premarital sex.

It is important to examine the psychosocial and demographic factors that predict premarital sexual practice among university students. Based on the theoretical literature and empirical findings discussed before, the following conceptual framework was developed. Some examples of studies related to this conceptual framework are (Anene et al., 2017) who found that adolescents with low self-esteem had higher risk of sexual behaviors. Barnes (2007) research indicated that negative peer influences on sexual activity were stronger for girls than boys. Ojedokun and Balogun (2008) reported that gender and premarital sexual permissiveness were strongly influenced by sex (Figure 2).

The aim of this study was to examine how self-esteem, peer pressure, and sex affect students’ attitude toward premarital sexual practice at Bahir Dar University. This research was designed to answer the following basic research questions.

- How self-esteem and peer pressure affect students’ premarital sex attitude?
- How do self-esteem and peer pressure differ among female and male students who had premarital sex?
- To what level the independent variables predict attitude toward premarital sex?

## 2 Methods

### 2.1 Study design, setting and population

At Bahir Dar University in Ethiopia, a correlational design was used with an institutional focus was carried out during February and March of 2022. The study population is made up of volunteers chosen at random, whereas all students enrolled in classes are referred to as the source population. Moreover, postgraduate, extension, and married students were not included in the study; only those registered as ordinary daytime, single students were.

### 2.2 Sample size determination

To determine the sample size a single population proportion formula, $n=\frac{z^{2}\;p\left(1-p\right)}{d^{2}}$ was used. *p* = 0.16 (16%) was used for participants expected to act properly toward premarital sexual practice by considering 95% confidence interval, marginal error (*d*) of 5 and 5% non-response rate.

Then, the calculated sample size would be:


$$n=\frac{z^{2}p\left(1-p\right)}{d^{2}}\mathrm{thus},n=\frac{{\left(1.96\right)}^{2}\;X\;0.16\;X\;1-0.16}{0.05^{2}}$$

= 206 + 5% Non-response rate and multiplying by 1.5 design effect 23 = 324, where *n* = Number of sample size *Z* = 95% confidence interval equals 1.96 *p* = 0.16 (16%), proportion of respondents expected to act properly toward pre marriage sexual practice. *d* = 0.05 (5%), marginal error. A multi-stage sampling technique was used to select 375 respondents for the study, although the analysis was based on the 361 questionnaires that were returned from the field and found useful for analysis.

### 2.3 Measurements

The researcher uses a variety of tools from other academics to evaluate psychosocial predictors of attitudes toward premarital sex among Bahir Dar University students. The Perception of Peer Pressure Scale, developed by Manzoni et al. (2011), was used to measure the prevalence of greater peer influence. The self-esteem measuring scale developed by Rosenberg (1965) was used to measure self-esteem, and the sexual attitude inventory scale developed by Eysenck and Eysenck (1971) was used to measure the intended behavior of the samples. The internal consistency of the Perception of Peer Pressure Scale was 0.83, while the self-esteem measuring scale had a reliability of 0.77. The internal consistency of the sexual attitude inventory scale was 0.97.

### 2.4 Data collection procedure

Initially, a letter of cooperation was obtained from the college and forwarded to the relevant authority. Participants and assistant data collectors received detailed orientations regarding the study’s aims. In addition, participants were informed that their information would be kept private and that they might withdraw at any time. Data were collected in person after the participants gave the researcher their written agreement. Eight qualified assistant data collectors in total were used to obtain pertinent information from participants.

### 2.5 Data analysis

The collected data was analyzed using mixed methods of analysis, which is a combination of quantitative and qualitative methods. The researchers used percentage, independent sample *t*-tests, and binary logistic regression to investigate numerical data. Phrases, words, and sentences were employed to examine qualitative data.

## 3 Major findings

### 3.1 Respondents background information

From the total population, 187 (51.80%) of the respondents were boys, and the remaining 174 (48.20%) were females. The majority of the participants were aged 15–20 years, and all the respondents were never married in their lifetime. The majority of them have one sexual partner and friend (Table 1). The age (mean ± SD) of the study participants was 17.4 ± 2.71 (15–24 years), 99.9 percent of the respondents were never married in their lifetime, and the majority of them have one sexual partner and friend.

**Table 1:** Level of self-esteem and exposure to peer pressure for premarital sex.

Variables	Observed mean	SD	Test value	Df	*T*	Sig. (2-tailed)
Self esteem	16.45	2.71	21.7	359	−43.33	0.000
Peer pressure	23.63	4.21	20	359	23.10	0.000

### 3.2 Experiences of premarital sexual practices

The average frequency of premarital sexual behavior was 48.6% [95% CI (44.31, 52.01%)]. 348 students, or 65.81 percent, had girlfriends or boyfriends. The majority of students (54.59%) who reported engaging in premarital sex were between the ages of 16 and 18. Of them, one hundred seventy-seven (67.86%) had sexual relations with their partner. For 114 (39.94%) of the respondents, peer pressure was the cause of their first sexual experience, while for 87 (37.63%), it was the desire to practice sex. Of the students who had sexual relationships, 131 (47.62%) did not utilize any form of contraceptive.

### 3.3 Level of self-esteem and exposure to peer pressure for premarital sex

The finding demonstrates that self-esteem, peer pressure, and demographic characteristics are key determinants of attitude toward premarital sexual conduct among students of Bahir Dar University in Ethiopia. According to the study, students who had engaged in premarital sex were more susceptible to peer pressure and had lower levels of self-esteem than their non-marital counterparts. According to the study, male students were more likely than female students to experience peer pressure and have higher levels of self-esteem. Moreover, the study indicated that self-esteem negatively influenced attitudes toward premarital sex, meaning that students with higher self-esteem were less likely to have a positive opinion of premarital sex. Students who have engaged in premarital sex had lower-than-average levels of self-esteem (*t* = −43.33, *p* = 0.000) and have been exposed to higher levels of peer pressure (*t* = 23.10, *p* = 0.000) (see Table 1).

### 3.4 Sex differences in level of self-esteem and exposure to peer pressure for premarital sex

The results of the research demonstrate that, in comparison to their female peers, male students who had engaged in premarital sex had greater levels of peer pressure and self-esteem. A report to Table 2 of the study, students’ attitudes toward premarital sex were also shown to be negatively associated with their self-esteem. This implies that those with higher self-esteem were less likely to have a positive perspective of the practice. Table 2 demonstrates a significant difference in self-esteem between men and women (mean = 15.76, SD = 2.77 and 14.61, SD = 2.01, respectively), *t* = 4.00, *p* = 0.000. Peer pressure exposure also differed significantly between males (mean = 28.13, SD = 4.79) and females (mean = 26.26, SD = 3.20; *t* = 5.91, *p* = 0.000). This indicates that compared to their female peers, male students who have engaged in premarital sex have higher levels of self-esteem and are more susceptible to peer pressure.

**Table 2:** Sex differences in level of self-esteem and exposure to peer pressure for premarital sex.

Variables	Sex	Mean	Sd	Df	*T*	Sig. (2-tailed)
Self esteem	Male	15.76	2.77	262.88	4.00	0.000
	Female	14.61	2.01			
Peer pressure	Male	28.13	4.79	257.09	5.91	0.000
	Female	25.30	3.20			

### 3.5 Attitude toward premarital sex on sex, self-esteem, and peer pressure

As shown in the Table 3, viewpoints among students regarding premarital sexual activity are significantly predicted by demographics, peer pressure, and self-esteem. The results of the study showed that students who had engaged in premarital sex were more susceptible to peer pressure and had lower levels of self-esteem. According to the findings of the study, male students were more exposed to peer pressure and had higher levels of self-esteem than female students. Moreover, the study indicated that self-esteem negatively influenced attitudes toward premarital sex, meaning that students with higher self-esteem were less likely to have a favorable view of premarital sex.

**Table 3:** Regression of attitude toward premarital sex on sex, self-esteem, and peer pressure.

Variables	B	S.E.	Wald	Df	Sig.	Exp (B)
Sex (1)	−0.133	0.330	0.160	1	0.701	0.778
Self-esteem	−0.144	0.072	5.300	1	0.023	0.871
Peer pressure	0.014	0.044	0.093	1	0.643	1.011
Constant	3.168	1.656	3.680	1	0.062	24.143

*χ2 = 9.360, df = 3, p = 0.00 Cox & Snell R Square = 0.030, Nagelkerke R Square = 0.033.*

The regression analysis showed that the three variables (sex, self-esteem, and peer pressure) were not statistically significant (*χ*2 (3, *N* = 361) =9.360, *p* = 0.00). Of the diversity in attitude toward premarital sex, the regression model only predicted 3.70% (Cox & Snell R Square) and 5.01% (Nagelkerke R Square). Eighty-six percent of the cases were correctly classified by the predictive algorithm (Table 3). Table 3 further reveals that, with a probability ratio of.778, self-esteem was the only variable that stood out as a unique and significant addition to the regression analysis. The findings indicate that there is a 64-fold decrease in respondents’ favorable attitudes about premarital sex for every unit rise in self-esteem. This indicates that opinions about premarital sex are negatively correlated with self-esteem.

## 4 Discussion

Different studies noted that 69.45% of women who practiced premarital sex expressed that they did so to upturn their self-esteem and 24% of women who had not intercourse assumed that they would have improved self-esteem if they participate in casual sex. The study also found that women’s sexual satisfaction was influenced by their marital status, marital quality, and marital communication (Ezeugwu and Ede, 2016; Weaver and Herold, 2000; Akililu et al., 2015). These studies indicate that there is a complex relationship between premarital sex and self-esteem, and that it may vary depending on individual factors such as gender, age, culture, education, and personal experiences.

The study suggests that students who have engaged in premarital sex have low self-esteem levels. This finding is related to Aleke et al. (2021). They found that adolescents who had low self-esteem were more likely to be exposed to premarital sexual behaviors. On the other hand, this finding is also in line with the work of Tasew’s (2011) who argued that low self-esteem leads individuals to engage in risky sexual behaviors such as unsafe sex, and having multiple sexual partners and sex before marriage. Similarly, the work of Aleke et al. (2021) also confirmed that low self-esteem is strongly linked to unsafe sexual behavior and premarital sex among adults.

This finding aligns with earlier studies (Winarni et al., 2016), which established a link between self-esteem and premarital sexual activity. It suggests that self-esteem tends to be higher in individuals who refrain from premarital sexual relations. Those with low self-esteem often feel inadequate, less competent, and less worthy compared to others.

This research indicates that university students who engage in premarital sex are often subject to increased peer pressure. This is consistent with Tasew’s (2011) findings, which suggest that peers of the same sex are a primary source of information related to sex and also provide the environment where sexual activities occur. By viewing sexually experienced friends as role models, same-sex peers can shape the perceived acceptability of sexual behavior. Similarly, Aleke et al. (2021) found that peers often replace adults as the main influencers of values, norms, culture, and behaviors, including sexual activities, among adolescents. This study corroborates the findings of previous research (Winarni et al., 2016; Anene et al., 2017), which established a link between peer pressure and premarital sexual behavior. Peers are seen as having a significant influence on adolescent sexual behavior within the realm of social dynamics. They serve as a crucial source of information about sex, significantly shaping the knowledge, attitudes, and sexual behaviors of adolescents.

This study discovered that self-esteem varies significantly between men and women in relation to sex, a finding that contradicts Habte et al. (2017) study which found that women with higher self-esteem had more sexual partners than those with lower self-esteem. The study also found a notable difference in the level of peer pressure experienced by men and women, with men being more affected. This is in contrast to Tasew’s (2011) assertion that women experience more negative peer pressure during sexual activity.

Another key finding of this study is that self-esteem significantly influences attitudes toward premarital sex. The data suggests that for each unit increase in self-esteem, respondents are 0.63 times less likely to view premarital sex favorably. This finding partially aligns with Weaver and Herold (2007) study, which revealed that 25% of women have engaged in unintended sex to boost their low self-esteem. Conversely, 24% of women who abstained from sex believed their self-esteem would improve if they participated in unintentional sexual relationships.

Contrary to some findings, this study aligns with research by Anyama (2019), which found that 24.4% of female participants engaged in premarital sex to feel “attractive,” a behavior that can be seen as a reflection of self-esteem. The act of participating in premarital sex can enhance a woman’s self-perceived value. Women, particularly those facing security issues or living alone, may engage in premarital sex to boost their self-esteem and form sexual partnerships (Yip et al., 2013; Richards, 2013).

In this study, gender did not predict attitudes toward premarital sex. This contrasts with previous studies like Habte et al. (2017), which found that male students had more permissive attitudes toward premarital sex than female students. Conversely, Abiy et al. (2018) found that women face harsher judgment than men for engaging in premarital sex, and as a result, women reported less casual sex than men.

Habte et al. (2017) found that living arrangements significantly influence attitudes toward premarital sex, with students living alone showing the most permissive attitudes. Those living with parents or guardians were least permissive. Similarly, Abiy et al. (2018) found that adolescents’ living conditions affect their sexual behavior. Tasew’s (2011) research in Ethiopia linked premature sexual intercourse to a lack of HIV/AIDS knowledge and substance use.

## 5 Conclusion and recommendation

The findings of the study showed that self-esteem and attitude toward premarital sexual relationships are modifiable factors influencing involvement in premarital sexual relationship among students. Due to the influence of negative peer pressure and the absence of parental oversight, students might exploit their personal freedom to engage in sexual activities. Based on this conclusion, the following recommendations were made:

- There is an urgent need to design an intervention to address the high level of premarital sexual relationships among students in the study area.
- There is an equal need for educational and therapeutic measures to help adolescents with low self-esteem since the findings of the study revealed that both constructs were significant predictors of premarital sexual relationships among the respondents.
- Intervention aimed at addressing poor attitudes toward premarital sex, as is the case in the developed world, must be given serious consideration in this context to help stem the tide of risky sexual behavior among young people and their ugly concomitants.
- There is a need to employ more counselors in universities and equip these counselors with all they need to ensure that sexual maladjustment among students in school is prevented and corrected.
- Teachers and parents alike must wake up to their responsibilities of monitoring and supervising their students and children, respectively. This is very important to reduce negative peer influences, which the study showed could predispose young people to premarital sexual relationships in school.

## Data Availability

The original contributions presented in the study are included in the article/supplementary material, further inquiries can be directed to the corresponding author.
